# Surface, Microstructural, and Mechanical Characterization of Prefabricated Pediatric Zirconia Crowns

**DOI:** 10.3390/ma12203280

**Published:** 2019-10-09

**Authors:** Valeria Diener, Georgrios Polychronis, Juliane Erb, Spiros Zinelis, Theodore Eliades

**Affiliations:** 1 Clinic of Orthodontics and Pediatric Dentistry, Centrer of Dental Medicine, University of Zurich, 8032 Zurich, Switzerland; valeria.diener@zzm.uzh.ch (V.D.); szinelis@dent.uoa.gr (S.Z.); 2 Department of Biomaterials, School of Dentistry, National and Kapodistrian University of Athens, 11527 Athens, Greece; gpolyg@yahoo.com; 3 In private practice, 8032 Zurich, Switzerland; juliane.erb@kinderzahnizuerich.ch

**Keywords:** zirconia, pediatric crowns, Raman, surface roughness, mechanical properties

## Abstract

The purpose of this study was to characterize the surface roughness, the microstructure, and mechanical properties of four prefabricated zirconia pediatric crowns. Ten prefabricated crowns from four different manufacturers (Cheng Crowns Zirconia), (EZCrowns), (NuSmile ZR), and (Zirconia Pediatric Crowns) were included in this study. The surface roughness parameters (Sa, Sq, Sz, Sc, and Sv) of all samples were studied by optical profilometry and then the microstructure was studied by Raman spectroscopy. Then, all samples were embedded in epoxy resin and after metallographic polishing, the Martens hardness (HM), indentation modulus (E_IT_), elastic index (η_IT_), Vickers hardness (HV), and fracture toughness (K_IC_) were identified by the Instrumented Indentation Testing (IIT). All data were statistically analyzed by a one-way ANOVA and a Tukey multiple comparison test at α = 0.05. Only the tetragonal phase of zirconia for all materials tested was identified after Raman analysis. However, statistically significant differences were found among the surface roughness parameters, HV and K_IC_, while no differences were allocated for HΜ, E_IT_, and η_ΙΤ_. Although the materials tested shared a similar microstructure, significant differences in surface roughness parameters HV and K_IC_ were identified and, thus, differences in their clinical performance were anticipated.

## 1. Introduction

Early Childhood Caries (ECC) represents the most prevalent childhood chronic disease [1,2] and is one of the leading causes of premature deciduous tooth loss [3]. The debilitating effects of tooth decay with regards to masticatory and speech function, as well as the deterioration of arch dimension stability, aesthetics, and quality of life, emphasizes the importance of effective treatment [4]. Besides the common restorative materials, like composite resins and amalgam, prefabricated pediatric crowns represent one treatment modality for extensive-multiface deciduous tooth carious lesions that cannot be treated with the former solutions [5].

Pediatric crowns should be able to withstand masticatory forces, show biocompatibility, facilitate oral hygiene [6], present high bonding strength, and not cause damage to the antagonist teeth. In addition, a high demand of aesthetics has been demonstrated to be one of the most critical issue in pediatric patients [7] and this has led manufacturers and clinicians to partially replace stainless steel crowns (SSC) by the recently introduced aesthetic ceramic ones [8]. Zirconia pediatric crowns (ZC) are considered to be a first choice in deciduous tooth restoration, which combines high strength, superior biocompatibility [8,9,10], improved wear resistance, and color stability, in contrast to the polycarbonate and composite or epoxy resin and thermoplastic pre-veneered stainless steel crowns [11,12,13]. The adoption of prefabricated zirconia crowns is considered to be a promising alternative in the restoration of primary teeth, combining clinically acceptable restorations [14,15] and fulfilling aesthetics demands [15]. A recently published clinical study has found out that zirconia crowns provide better gingival health and less plaque accumulation, compared to SSC ones [16].

However, the implementation of prefabricated zirconia crowns is not free of limitations and drawbacks. The latter might require a greater amount of tooth reduction [17,18], whereas, their increased hardness might lead to tooth wear of the antagonist teeth [13]. Finally, bond strength and surface alteration still constitutes a challenge [19,20]. Furthermore, Zirconia is a polymorphic material which requires the presence of stabilizers, like yttrium and magnesium oxide, to prevent tetragonal/cubic phases to swing to the monoclinic one at room temperature, characterized by inferior mechanical properties [21]. Even small deviations of the amount, as well as the type of phase stabilizer, might have definite effects on phase consistency-crystal structure and consequently on mechanical, thermal, and electrical properties [21,22]. In addition, manufacturing processes might further affect the material structure and surface characteristics by introducing cracks, modulating roughness, and determining the grain size [22], which in turn influence crown compressive strength, fracture toughness, hardness, aesthetics, plaque retention, and bonding strength. Consequently, zirconia-made pediatric crowns differing in chemical synthesis-microstructure and production method parameters might show a wide range of mechanical properties and inevitably an altered clinical behavior. Recently published studies identified differences in microleakage [23] surface roughness parameters [24], and fracture resistance [25] among different brands of prefabricated zirconia crowns.

Therefore, the aim of this study was to compare the surface roughness, microstructure, and mechanical properties of zirconia-made pediatric crowns from four different manufacturers. The null hypothesis set was that all materials share equal surface roughness characteristic, microstructure, and mechanical properties.

## 2. Materials and Methods

### 2.1. Materials

Forty prefabricated molar crowns from four different manufacturers were included in this study. The brand names, the manufacturer, and the code of materials tested are presented in Table 1.

### 2.2. Optical Profilometry

The surface analyses were done with optical profilometry. The surface roughness of each group (IE, SB, AE, and SBAE) was examined at 5 regions per specimen with an optical interferometric profiler (Wyko NT1100; Veeco, Tucson, Ariz) operated under the following conditions: Mirau 20-times objective lens, vertical scanning mode, 15-μm vertical scan length, 231.1 × 303.8 μm analysis area and tilt corrections with 0.1-nm (z-axis) and 0.2-μm (x-axis and y-axis) resolutions. The surface roughness parameters measured were the amplitude parameters Sa (arithmetic mean deviation), Sq (root mean square roughness), and Sz (the maximum height of the surface), and two functional parameters Sc (core void volume) and Sv (surface void volume). The former indicated the volume (e.g., of a fluid filling the core) that the surface would support for 10%–80% of the bearing ratio and the latter from 80% to 100% of the bearing ratio.

### 2.3. Raman Microscopy 

After optical profilometry, one Raman spectrum was acquired from the occlusal region of all tested crowns. The crowns were placed on the stage of a microscope (LEICA BME, Leica microsystems Ltd., Heerbrugg, Switzerland) and the region of interest were determined by employing optical lens at 10× nominal magnification. The Raman probe was attached to the microscope employing a special device (MicroViewer-785, Raman Microscope Adaptor, TSI Inc, Shoreview, MN, USA). The spectra were acquired with EZ Raman-I, (High sensitivity portable Raman analyzer, TSI Inc, Shoreview, MN, USA) equipped with a Laser (Soliton, Laser Und Messtechnik, Gliching, Germany) operating at 450 mW output power, emitted wavelength 785 nm, and nominal resolution 4.5–6.5 cm^−1^.

### 2.4. Instrumented Indentation Testing (IIT) and Fracture Toughness

All crowns were embedded in the epoxy resin and subjected to metallographic grinding under continuous water cooling, until the circumferential of cervical region was exposed completely. Then, the samples were polished with 6, 3, and 1 μm diamond pastes (DP, Struers, Bellarup, Denmark). Both grinding and polishing were carried out in a grinding/polishing machine (Dap V, Struers). Then, the mechanical properties of all groups were identified by employing instrumented indentation testing (IIT), according to ISO 14577-2002 [26].

The mechanical properties tested were Martens Hardness (HM), indentation modulus (E_IT_), elastic index (η_IT_), Vickers Hardness (HV), and fracture toughness (K_IC_). The first three properties were determined according the formulas provided by the ISO 14577-2002. HV and K_IC_ were determined by measuring the diagonal of impression and the cracks, with the coupled optical measuring microscope at 20× nominal magnification. The optical microscope was mounted in a displacement unit designed to allow lenses and loading unit to exchange positions, and thus, specimen did not need to be relocated for optical measurements. HV was calculated directly by the software, while K_IC_ was calculated according to the Lankford formula [27]:

$$K_{IC}=0.0782\times \left(HV\times a^{0.5}\right)\times {\left(\frac{E}{HV}\right)}^{0.4\;}\times {\left(\frac{c}{a}\right)}^{-1.56}$$

where *HV* is Vickers Hardness (GPa), *E* stands for Young’s modulus (GPa), *a* is the half diagonal length of the indentation (m), and c is the crack length from the center of the indentation to the crack tip (m). 

### 2.5. Statistical Analysis

The results of surface roughness parameters (Sa, Sq, Sz, Sc, and Sv) and the mechanical properties (HM, E_IT_, η_IT_, HV and K_IC_) were statistically analyzed by one-way ANOVA, by employing the brand as the discriminating variable. The significant differences among groups were identified by the Tukey multiple comparison test at α = 0.05. The normality and homoscedasticity of all data were initially checked by the Kolmogorov–Smirnov test and the equal test variance, respectively. 

## 3. Results

### 3.1. Optical Profilometry

**Figure 1:**
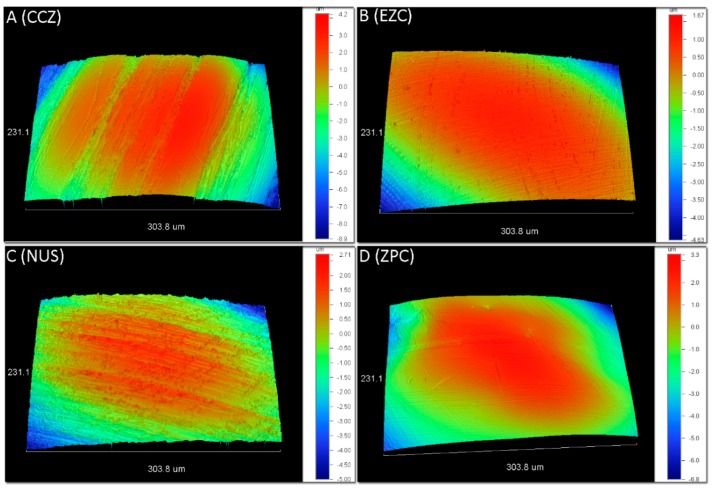
Representative 3D-optical profilometric images from all tested groups: (**A**) CCZ, (**B**) EZC, (**C**) NUS, and (**D**) ZPC. All surfaces demonstrate parallel striations and machining tracks. Please note the differences in scales.

Figure 1 demonstrates representative 3D profilometric images from the occlusal surfaces of all materials tested. All surfaces illustrated the presence of parallel striations. The results for surface roughness parameters and statistical outcome are presented in Table 2. For all amplitude parameters (Sa, Sq, Sz) the CCZ showed significantly higher values, compared to EZC. For both functional parameters (Sc and Sv), the CCZ depicted significant differences compared to EZC and ZPC. 

### 3.2. Raman Microscopy

**Figure 2:**
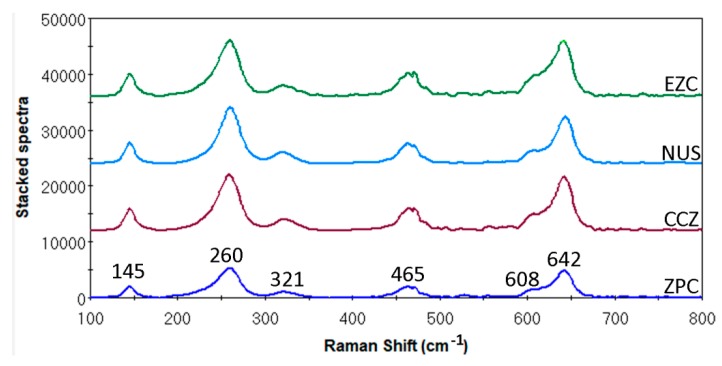
Representative Raman spectra from all tested materials.

Figure 2 demonstrates the representative Raman spectra from all tested materials. All materials showed identical Raman spectra. The characteristic peaks at 145, 260, 321, 465, 608, and 642 belonged to the tetragonal zirconia phase.

### 3.3. Instrumented Indentation Testing (IIT) and Fracture Toughness

**Figure 3:**
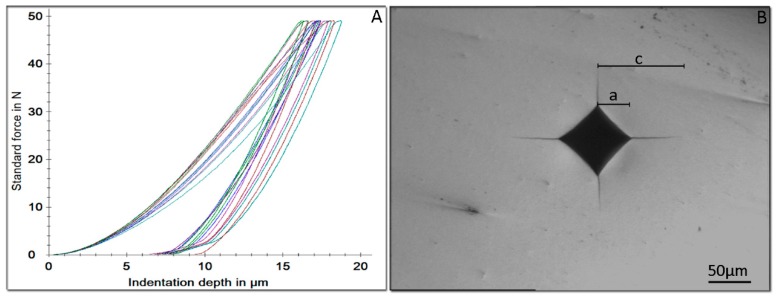
(**A**) Representative force indentation depth curves of the loading/unloading cycle from all tested materials. (**B**) Representative image of the Vickers impression from the surface of all tested materials. All cracks originated from the four corners of the Vickers impression. **a**—the half diagonal; and **c**—the distance from the center of the impression to the tip of the crack (bar: 50 μm).

In Figure 3, representative force indentation depth curves of the loading/unloading cycle from all tested materials are depicted. Table 3 demonstrates the mechanical properties (HM, E_IT_, η_ΙΤ_, HV, and K_IC_) of the tested materials. Significant differences were allocated only for HV and K_IC_ with NUS and ZNP showing significantly lower HV compared to the other two materials and EZC demonstrating significantly lower K_IC_, compared to the rest of the materials.

## 4. Discussion

Based on the results of this study the null hypothesis was rejected, as significant differences were identified among zirconia crowns in surface roughness parameters and the tested mechanical properties.

Contrary to Ra, which is measured over a line, Sa is a two dimensional measurement and thus the results of surface roughness parameters are not directly comparable with previously published data, especially among surfaces with varying textures such as zirconia crowns with oriented parallel striations. [24,28]. All crowns tested demonstrated these patterns (Figure 1) of oriented parallel striations, which should be appended to milling of crowns from prefabricated zirconia blocks. The significantly lower amplitude parameters (Sa, Sq, Sz) of EZC compared to CCZ might be appended to different milling parameters during the manufacturing process. Generally, higher roughness increases the enamel wear of opposite teeth and, thus, smoother surfaces are desirable [29]. In addition, EZC showed the lowest core (Sc) and surface void (Sv) volume parameters, compared to CCZ, denoting that it is less vulnerable to integuments retention and thus more resistance to discoloration during intraoral ageing. However, clinical data are required to verify this approach. 

Zirconia appears in three different crystallographic structures—the cubic (c), the tetragonal (t), and the monoclinic phase (m). Although the (t) phase is stable above 1170 °C, it can be stabilized at room temperature by the addition of 2~3% mole Yttrium oxide (Y_2_O_3_). The stabilization of (t) phase at room temperatures, combines an array of favorable mechanical properties with a bending strength above 1000 MPa and a fracture toughness of about 6 MPa m^1/2^ [30], and it is commonly known as the Yttria-stabilized Tetragonal Zirconia Polycrystal (Y-TZP). Raman spectroscopy is extensively used in Y-TZP Zirconia characterization as (t) and (m) phases can be easily distinguished. All tested materials showed identical spectra (Figure 2) with only the characteristic peaks of the (t) phase being presented [31]. This strengthened the assumption that the tested zirconia crowns were made of typical Y-TZP dental zirconia. In addition, as the monoclinic phase was not identified, it implied that the materials used were not destabilized by the manufacturing or storage processes [32]. Although the presence of the (c) phase was not expected in Y-TZP, its presence was hardly distinguished in the Raman analysis, due to the fact that (t) and (c) share overlapping characteristic peaks [31].

No statistically significant differences were identified among the tested materials after IIT, denoting that all materials shared equal HM, elastic modulus, and elastic index. There is no data for HM in dental literature and thus no comparison could be made with previously published data. On the contrary, the elastic modulus (E_IT_) was found to almost half the nominal value of zirconia (210 GPa) [33]. This inconsistency was a known complication in IIT, when non-stress-free sample were investigated. It is well-known that samples with tensile residual stresses showed decreased elastic modulus, while those with residual compressive stresses demonstrated a higher modulus [34]. Therefore, it might be concluded that pediatric crowns are delivered with an unknown extent of residual stresses, which are probably induced during the manufacturing processes. Elastic index is indicative of the brittleness of the materials tested and according to the results, no differences were identified among the materials tested for this property. Although both expressions of hardness (HM and HV) showed the same classification of materials tested, only in Vickers was a significant difference noted, and this might be assigned to the residual stresses which affect the elastic rebound around indentation, after force removal. It is noteworthy that HM was automatically calculated by maximum force and penetration depth during testing, while HV was evaluated after force removal and the final diagonal size and, thus, effect such as creep, elastic rebound, perception of operator, and others also interfered [35]. However, the measurement of HV was essential in this experiment for the evaluation of K_IC_. Vickers Hardness were found to be close to previously published data 1367–1368 [36], and 1344–1379 [33] for Y-TZP dental zirconia. 

The most important clinical implication of HV is the wear imposed on the antagonist teeth as it is considered that higher hardness of restorative materials results in more enamel wear [37]. The clinical implications of these findings are two-fold. First, dentists should consider that primary teeth are more prone to wear than permanent dentition and secondly that a permanent teeth might undergo extensive wear from an antagonist temporarily restored by prefabricated pediatric zirconia crown. However the enamel loss of primary teeth due to wear from a zirconia crown has not been reported yet and, therefore, no clinical guidelines could be derived at this point. Therefore, based on the aforementioned approach, NUS and ZPC with lower HV values were expected to perform better clinically. The estimation of K_IC_ with indentation methods has a long record with different approaches and mathematical formulas. Although these formulas are divided on the basis of the Palmqvist or median cracks, a recent study has pointed out that the Lankford formula which is used in both crack systems, yield values that are quite similar to the values for Y-TZP zirconia found in literature, which was the rational for selecting the Lankford formula in this study. [33]. Fracture toughness is indicative of the resistance of materials to crack propagation and, thus, the clinical implication is that materials with lower K_IC_ might be more prone to cracking and chipping, especially at the thinner cervical regions of prefabricate zirconia crowns. In a recent study, crowns made of zirconia were found to be more prone to fracture, compared to those made by metallic alloys [38]. Therefore, EZC with significantly lower K_IC_ might be more vulnerable to crack initiation and propagation under in-service conditions.

## 5. Conclusions

Raman, Vickers, and fracture toughness results showed that pediatric zirconia crowns are made of Y-TZP zirconia. However, that prefabricated zirconia crowns showed significant differences in surface roughness parameters, HV and K_IC_, and thus differences in their clinical performance are anticipated.

## Figures and Tables

**Table 1:** Brand name, manufacturer, and code of all materials tested (n = 10).

Brand Name	Manufacturer	Code
Cheng Crowns Zirconia	Cheng Crowns Exton, PA, USA	CCZ
EZCrowns	Sprig Suite L Loomis, CA, USA	EZC
NuSmile ZR	NuSmile, Houston, TX, USA	NUS
Zirconia Pediatric Crowns	Kinder Krowns, St Louis Park, MN, USA	ZPC

**Table 2:** Mean values and standard deviations in parentheses along with the statistical outcome of all tested groups.

Group	Sa (μm)	Sq (μm)	Sz (μm)	Sc (μm^3^/μm^2^)	Sv (nm^3^/nm^2^)
CCZ	2.7 (0.7) ^a^	3.4 (1.0) ^a^	18.7 (6.5) ^a^	3.1 (0.6) ^a^	563 (184) ^a^
EZC	1.1 (0.2) ^b^	1.4 (0.3) ^b^	8.2 (1.4) ^b^	1.3 (0.1) ^b^	265 (62) ^b^
NUS	1.7 (0.5) ^ab^	2.2 (0.7) ^ab^	11.3 (2.6) ^ab^	2.2 (0.7) ^ab^	265 (50) ^ab^
ZPC	1.6 (1.0) ^ab^	1.9 (1.2) ^ab^	9.6 (6.2) ^ab^	1.7 (0.8) ^b^	188 (53) ^b^

Same superscripts connect mean values without statistical significant differences.

**Table 3:** Mean values and standard deviations (in parentheses) of the tested mechanical properties.

Material	HM (N/mm^2^)	E_IT_ (GPa)	n_IT_ (%)	HV_5_	K_IC_ (MPa m^1/2^)
CCZ	6653 (635) ^a^	123.1 (15.7) ^a^	52.0 (4.5) ^a^	1365 (20) ^a^	6.1 (0.3) ^a^
EZC	6390 (584) ^a^	118.1 (12.4) ^a^	54.4 (1.6) ^a^	1347 (12) ^a^	4.7 (0.3) ^b^
NUS	6142 (430) ^a^	114.3 (13.9) ^a^	54.9 (5.3) ^a^	1305 (18) ^b^	8.0 (1.8) ^a^
ZPC	6318 (402) ^a^	114.2 (13.7) ^a^	53.3 (2.9) ^a^	1325 (14) ^b^	6.0 (0.5) ^a^

Same superscripts connects mean values without statistical significant differences (*p* > 0.05).
